# Supplementary material for: Transcriptomics and Functional Analysis of Copper Stress Response in the Sulfate-Reducing Bacterium Desulfovibrio alaskensis G20
Source: Int J Mol Sci. 2022 Jan 26;23(3):1396. doi: 10.3390/ijms23031396 (PMC8836040; doi:10.3390/ijms23031396)
Supplement: Supplementary file 1 [file ijms-23-01396-s001.zip › Supplementary_Information.pdf]

International Journal of
Molecular Sciences

MDPI

Table S1: Lactate-C media composition

Component	Amount (g/L)
Sodium lactate	6.8
Sodium sulfate	4.5
Calcium chloride dehydrated	0.06
Sodium citrate	0.3
Ammonium chloride	1
Potassium phosphate monobasic	0.5
Yeast extract	1
Ascorbic acid	0.1
Sodium thioglycolate	0.1

Table S2: List of all genes and primer sequences used for RT-qPCR validation

Gene	Forward Primer	Reverse Primer	Source
Dde_3061	TTTTCATCGCTGCCAAAGGC	CCGCCGTAGTAGTGGGTTTT	This study
Dde_3729	CACTGCTGCGCTGTATCAAC	AACACCCTGTCAGACGTTCC	This study
Dde_1264	CAAGGAACTGTTTGCCCGTG	CCAGCTCGAATTTGCCGATG	This study
Dde_3047	CCGTGGGTGGTGATTCCTAC	AGGCGCTCAAAAAGCGAATG	This study
Dde_0356	AAGCGCCAGAACGTCATCAT	TTCCGTACCCAGCGAAAGTG	This study
Dde_0347	GAGATAGCCGAGCAGGTGTC	AGAATTACGCGCCACTTCCA	This study
Dde_1454	CGGTGAAATTGTAACCGGCG	CGAACAGAATGCGGTCGATG	This study
Dde_1415	TGAAATCGCAGGAAGAGCGT	CGGCAATGCGTCTGTAGTTG	This study
Dde_1602	GTATTTCACCCGGCAGTCCA	AAAAAGCGGAGCTGCAATGG	This study
Dde_0975	TTCCGCAAATTTGAGCAGGC	CGGCAGAACAGTGGTTACCT	This study
Dde_1800	CCGGAACACACCATGTACGA	ACAGGCAGCAGACGATGAAA	This study
Dde_2100	TCAGGGTGCTGCTGCATAAT	TTTCTGCTACTTCGGCTGTGT	This study
Dde_3775	AATGTGTAGACGTGTGCCCC	ACTTCGACGCAGGATTCACA	This study
Dde_2561	AGAAGCCGCGGAATTTCTGA	TCTTTCCAGTTCGTCCAGCC	This study
Dde_3201	GACGGCAAATGGCAGATACG	CGTACGGTGGTCACCAGTTT	This study

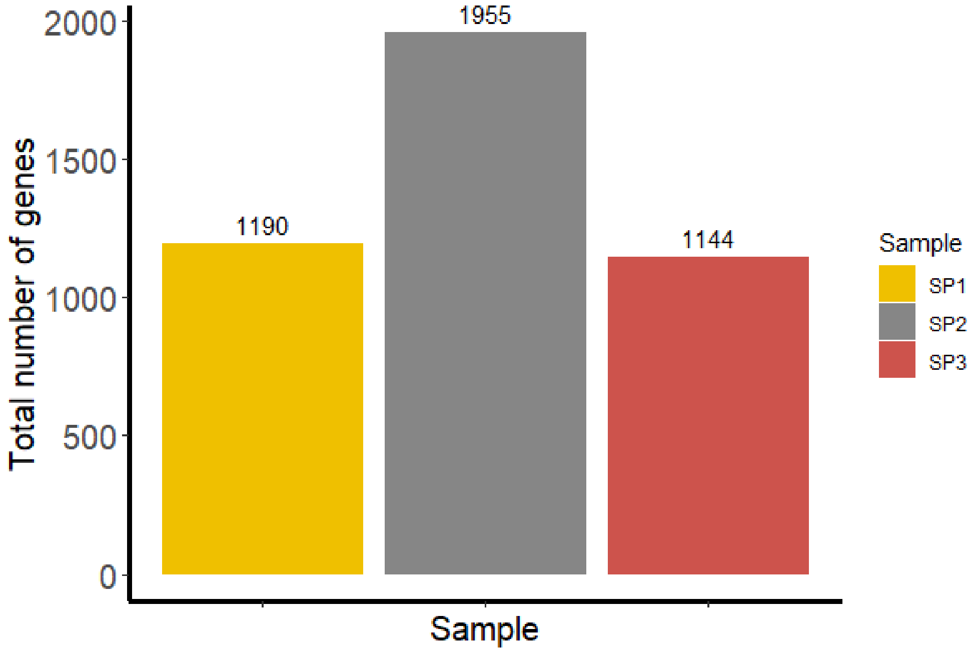

Figure S1: Total number of DEGs in all the samples (SP1: 0 μM vs. 5 μM Cu(II); SP2: 0 μM vs. 15 μM Cu(II); SP3: 5 μM vs. 15 μM Cu(II))(p-value < 0.05 and $|\log_2(FC)| > 0$).

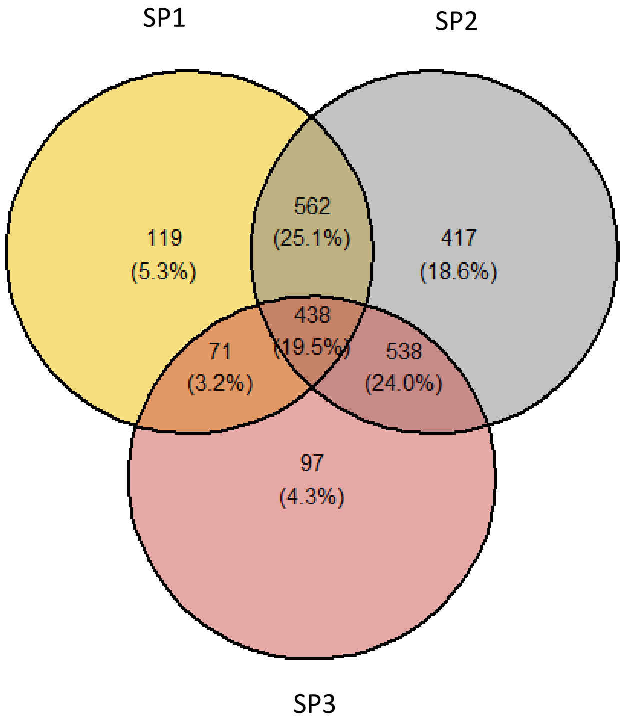

Figure S2: Venn diagram showing percentage of genes shared between all three sample pairs. (SP1: 0μM vs. 5μM Cu(II); SP2: 0μM vs. 15μM Cu(II); SP3: 5μM vs. 15μM Cu(II))

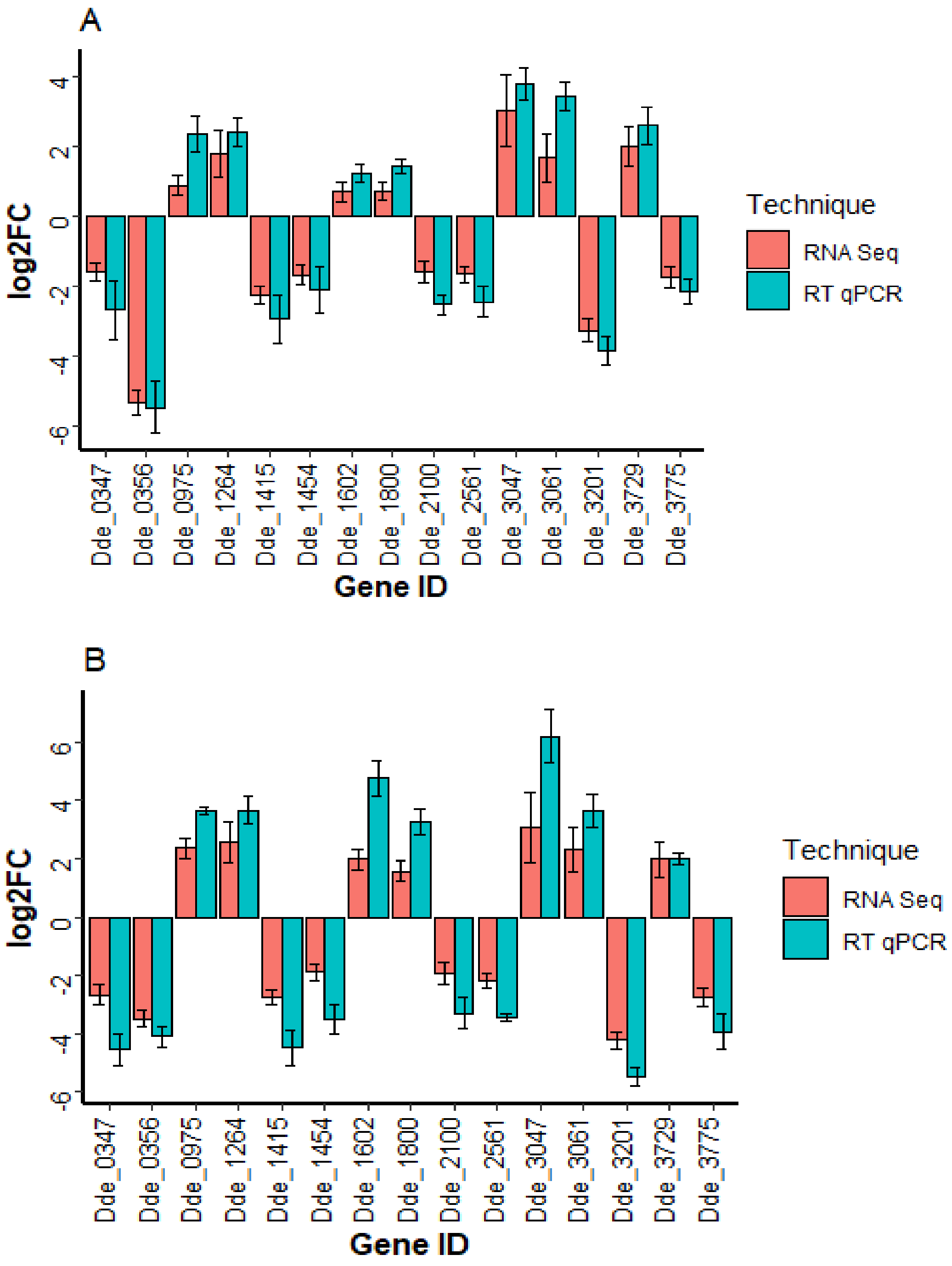

Figure S3: RT-qPCR validation of RNA-Seq data for (A) 0µM vs 5µm Cu(II) and (B) 0µM vs 5µm Cu(II).

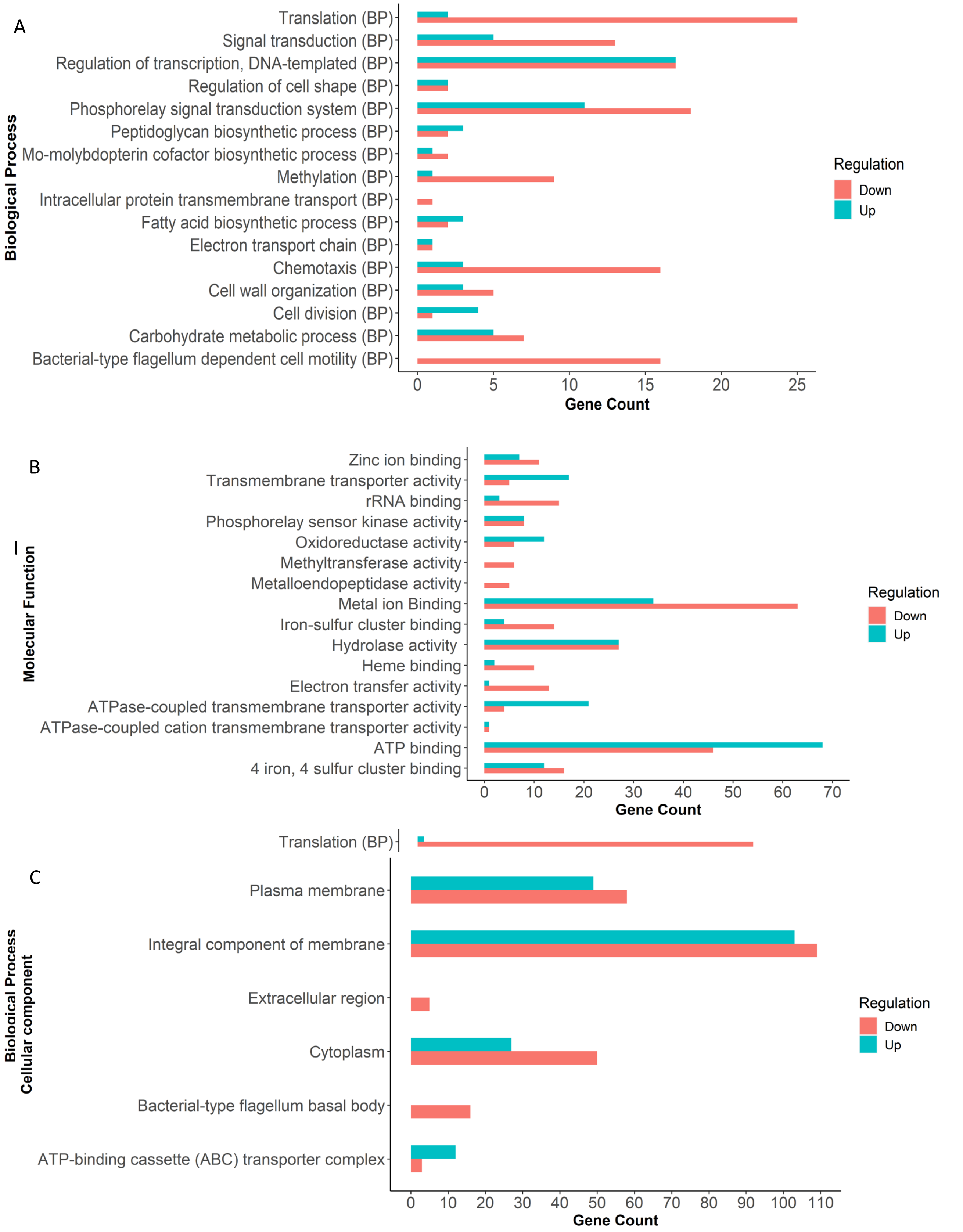
A
Biological Process
Translation (BP)
Signal transduction (BP)
Regulation of transcription, DNA-templated (BP)
Regulation of cell shape (BP)
Phosphorelay signal transduction system (BP)
Peptidoglycan biosynthetic process (BP)
Mo-molybdopterin cofactor biosynthetic process (BP)
Methylation (BP)
Intracellular protein transmembrane transport (BP)
Fatty acid biosynthetic process (BP)
Electron transport chain (BP)
Chemotaxis (BP)
Cell wall organization (BP)
Cell division (BP)
Carbohydrate metabolic process (BP)
Bacterial-type flagellum dependent cell motility (BP)
0 5 10 15 20 25
Gene Count
Regulation
Down
Up
B
Molecular Function
Zinc ion binding
Transmembrane transporter activity
rRNA binding
Phosphorelay sensor kinase activity
Oxidoreductase activity
Methyltransferase activity
Metalloendopeptidase activity
Metal ion Binding
Iron-sulfur cluster binding
Hydrolase activity
Heme binding
Electron transfer activity
ATPase-coupled transmembrane transporter activity
ATPase-coupled cation transmembrane transporter activity
ATP binding
4 iron, 4 sulfur cluster binding
0 10 20 30 40 50 60 70
Gene Count
Regulation
Down
Up
Translation (BP)
C
Biological Process
Cellular component
Plasma membrane
Integral component of membrane
Extracellular region
Cytoplasm
Bacterial-type flagellum basal body
ATP-binding cassette (ABC) transporter complex
0 10 20 30 40 50 60 70 80 90 100 110
Gene Count
Regulation
Down
Up

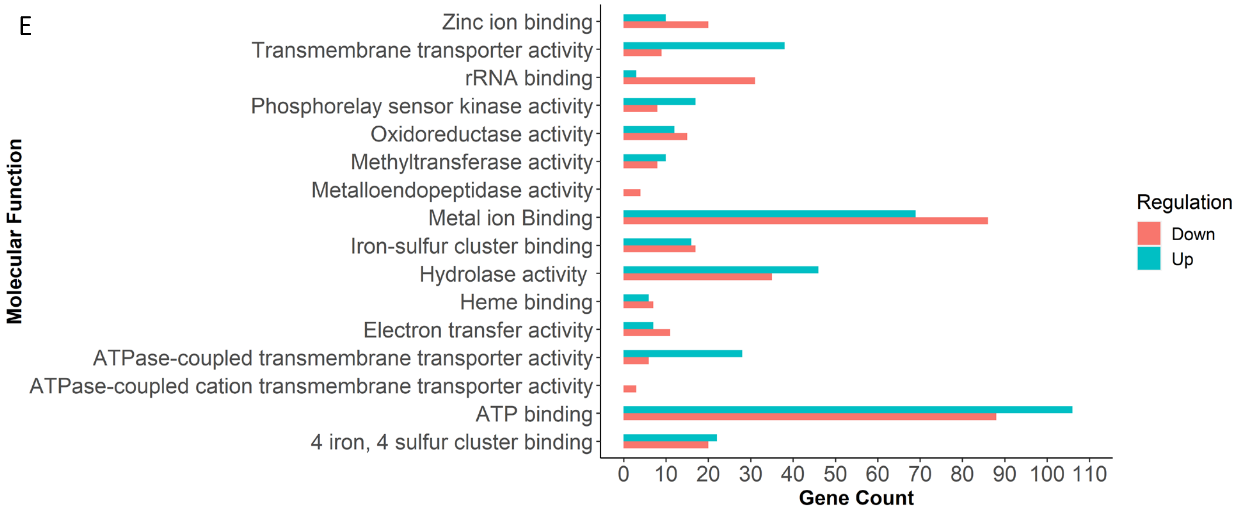

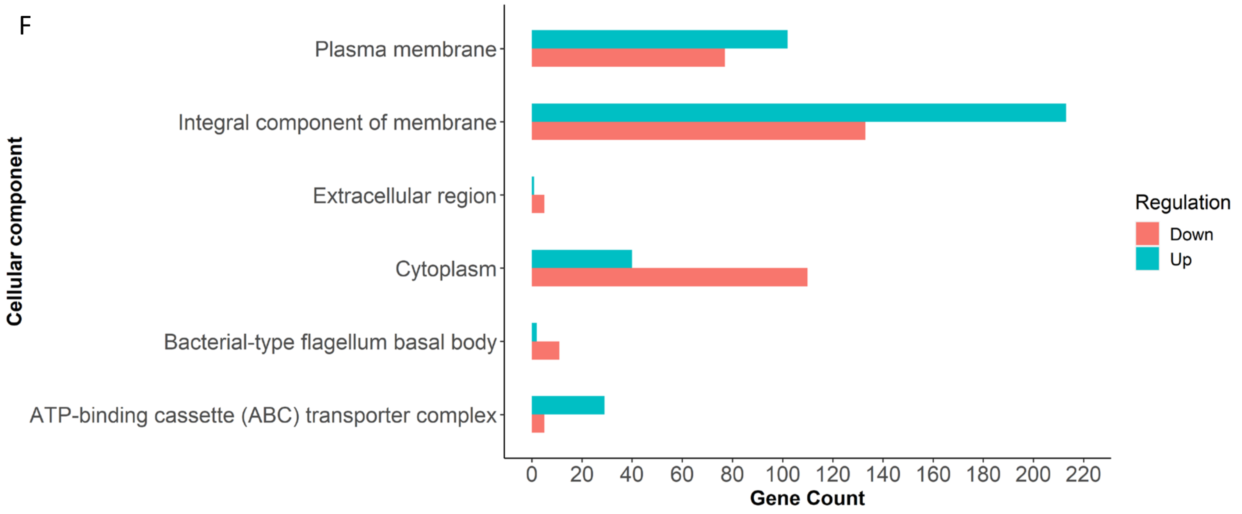

Figure S4: Bar plot of depicting the number of upregulated and downregulated genes in (A) BP (0μM Cu(II) vs 5μM Cu(II); (B) MF (0μM vs 5μM Cu(II)); (C) CC (0μM Cu(II) vs 5μM Cu(II)); (D) BP (0μM Cu(II) vs 15μM Cu(II)); (E) MF (0μM Cu(II) vs 5μM Cu(II)); (F) CC (0μM Cu(II) vs 15μM Cu(II)).

GC/MS data transformation and analysis:

GC/MS spectra data from metabolomic analyses were transformed as follows: i) removal of metabolites with >25% of missing data (no spectra); ii) data with <25% missing values were replaced with ½ of observed minimum positive detection value, iii) data were normalized by summing of the spectral signals obtained across all metabolites for a given library (row sum) and log transformation, and iv) data were mean centered and divided by the standard deviation of each variable. The DeviumWeb interface in R was used for correspondence analysis for metabolomic characterization. Multiple comparisons were controlled for false discovery rate (FDR).
