# Supplementary figures and images for: Transcriptomics and Functional Analysis of Copper Stress Response in the Sulfate-Reducing Bacterium Desulfovibrio alaskensis G20
Source: Int J Mol Sci. 2022 Jan 26;23(3):1396. doi: 10.3390/ijms23031396 (PMC8836040; doi:10.3390/ijms23031396)

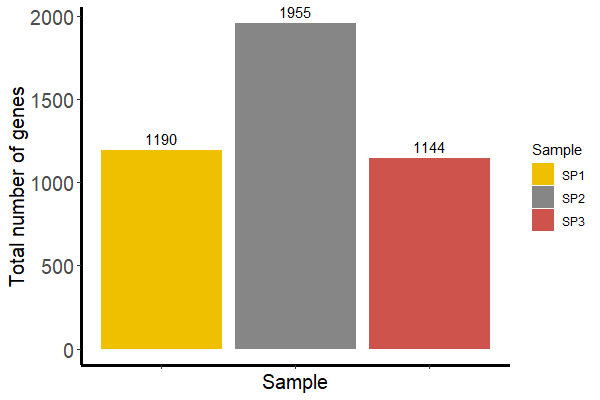

Supplement: Supplementary file 1 [file ijms-23-01396-s001.zip › Figure S1.png]

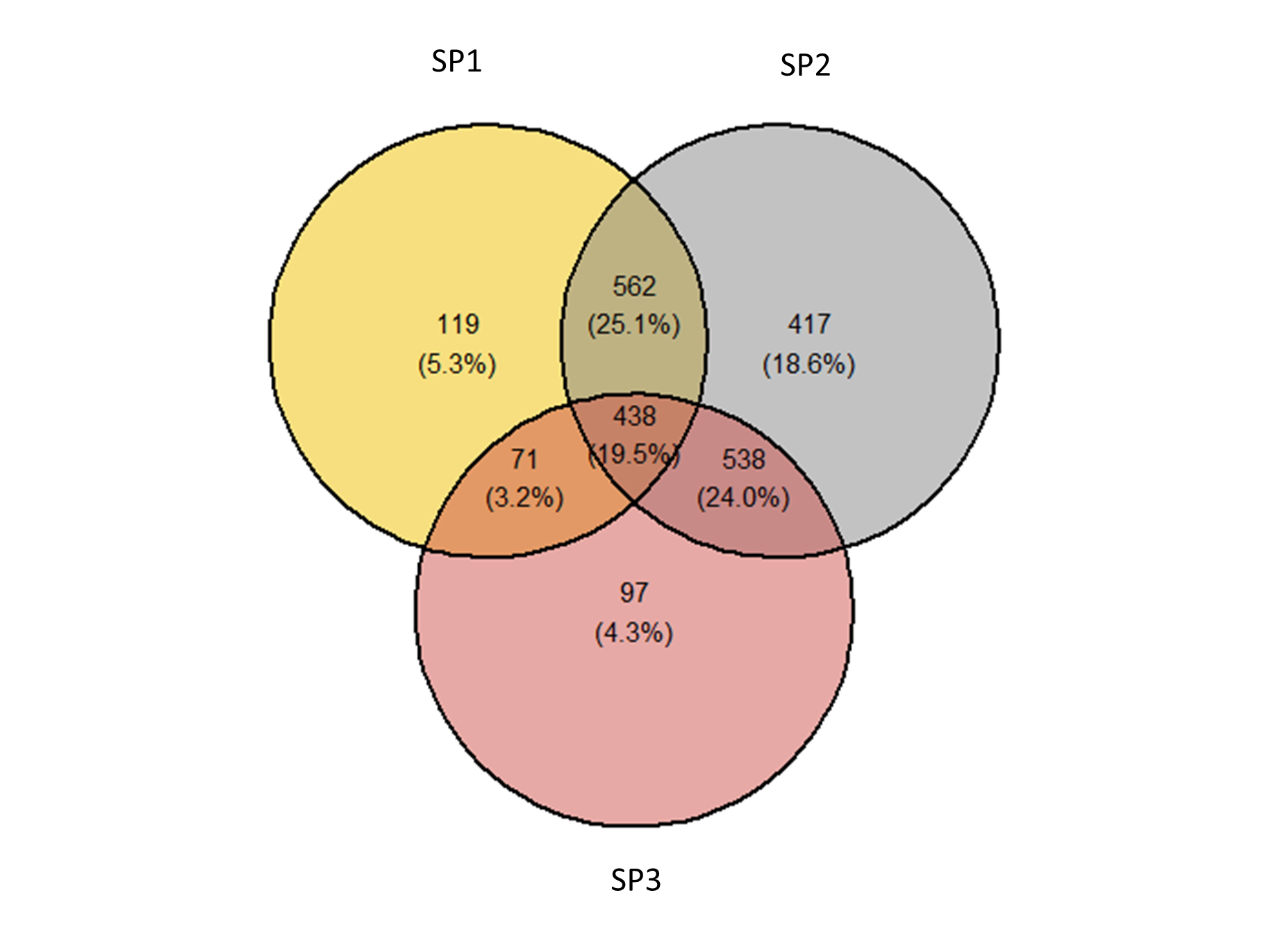

Supplement: Supplementary file 1 [file ijms-23-01396-s001.zip › Figure S2.png]

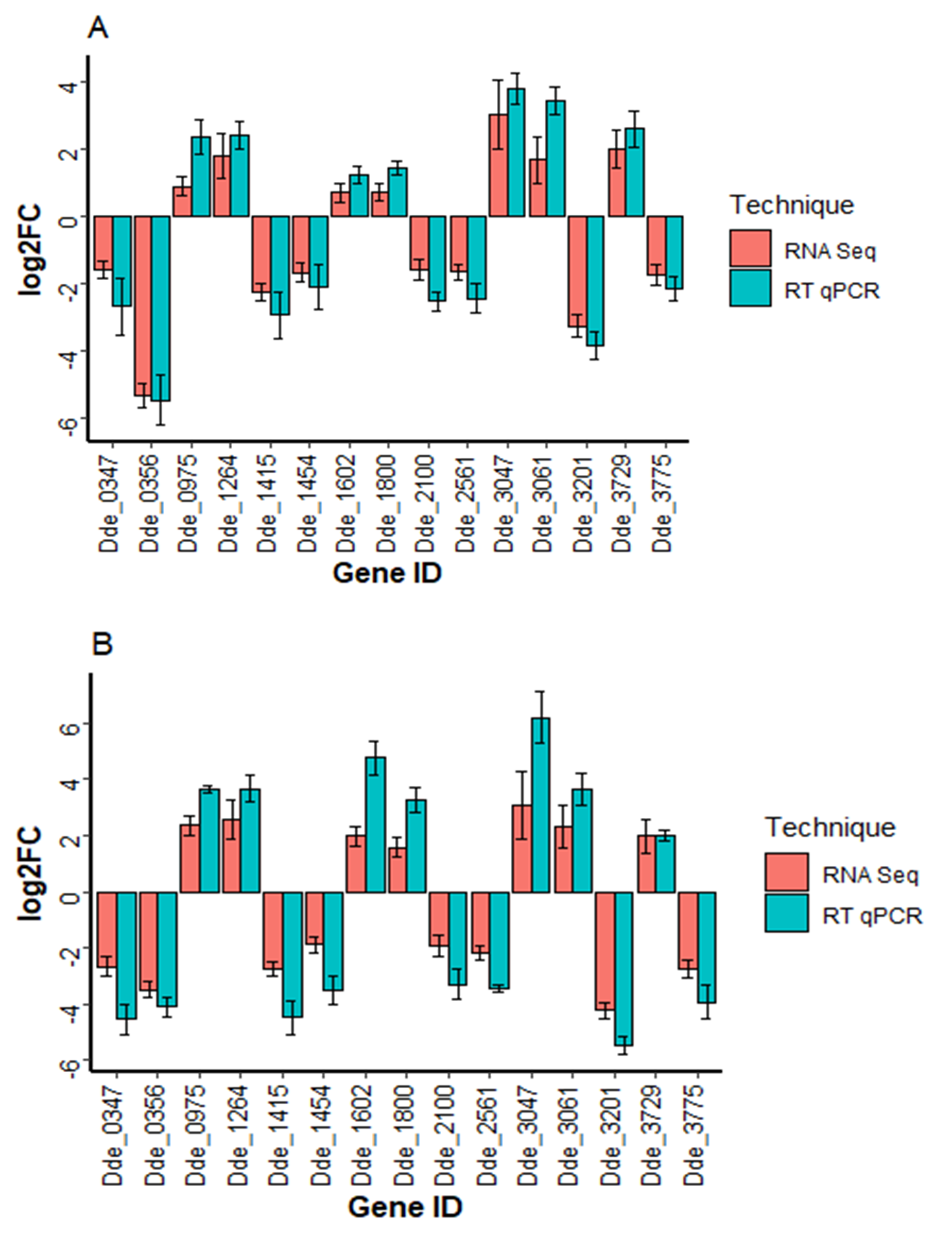

Supplement: Supplementary file 1 [file ijms-23-01396-s001.zip › Figure S3.png]

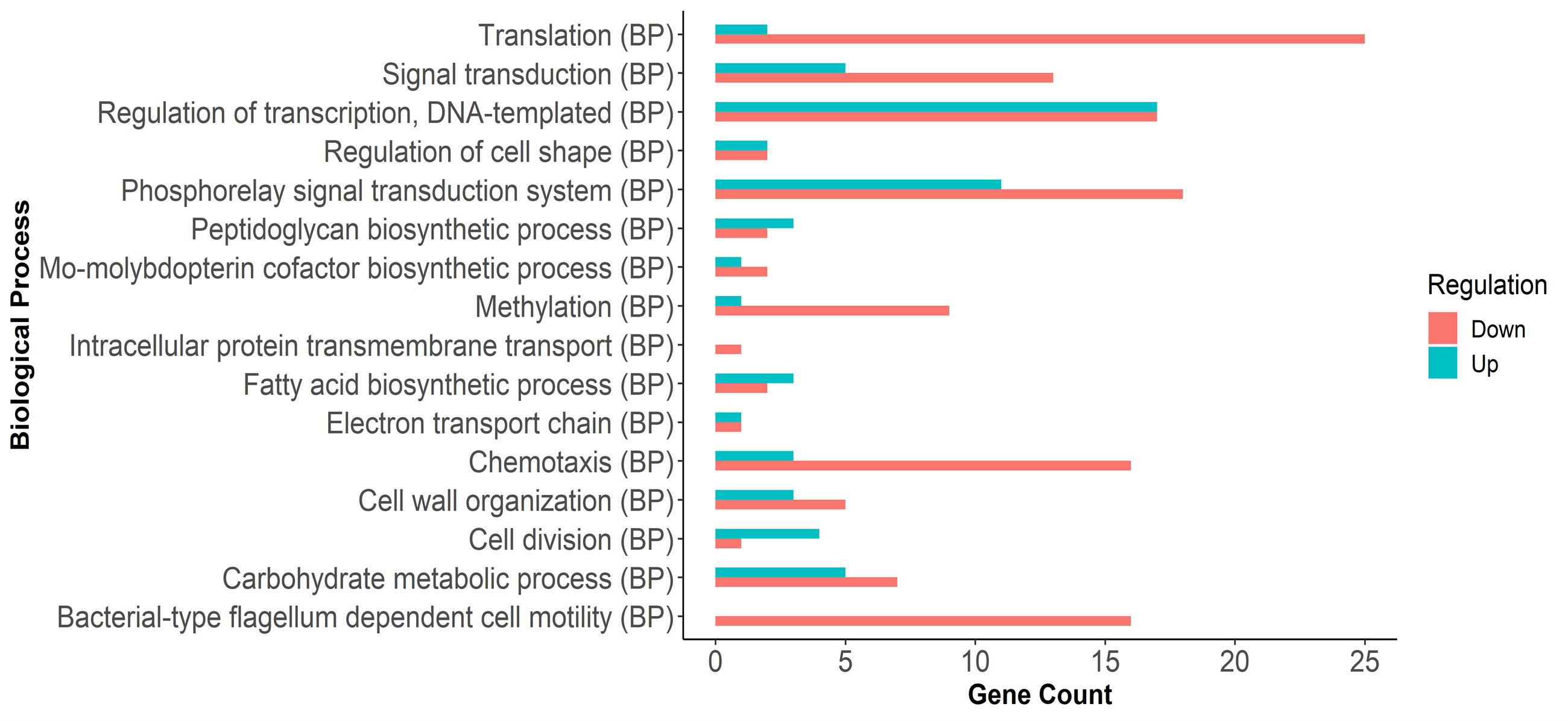

Supplement: Supplementary file 1 [file ijms-23-01396-s001.zip › Figure S4A.png]

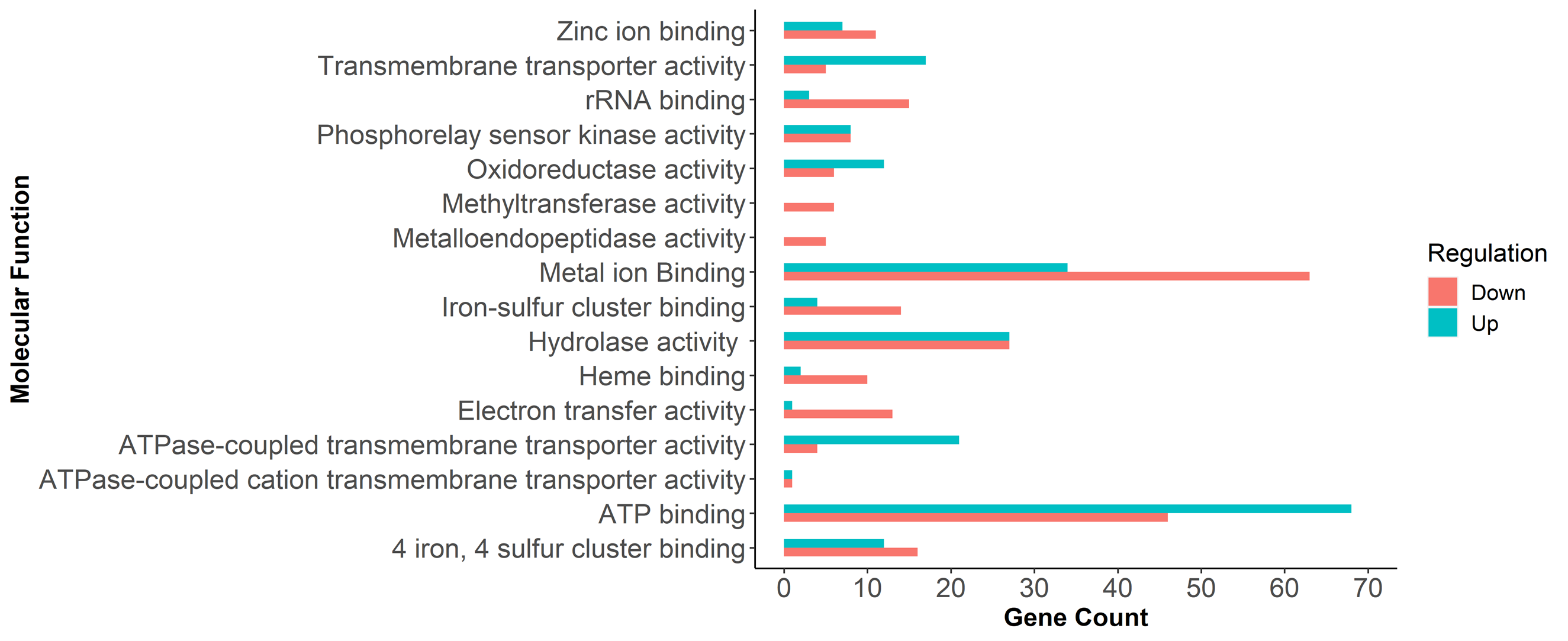

Supplement: Supplementary file 1 [file ijms-23-01396-s001.zip › Figure S4B.png]

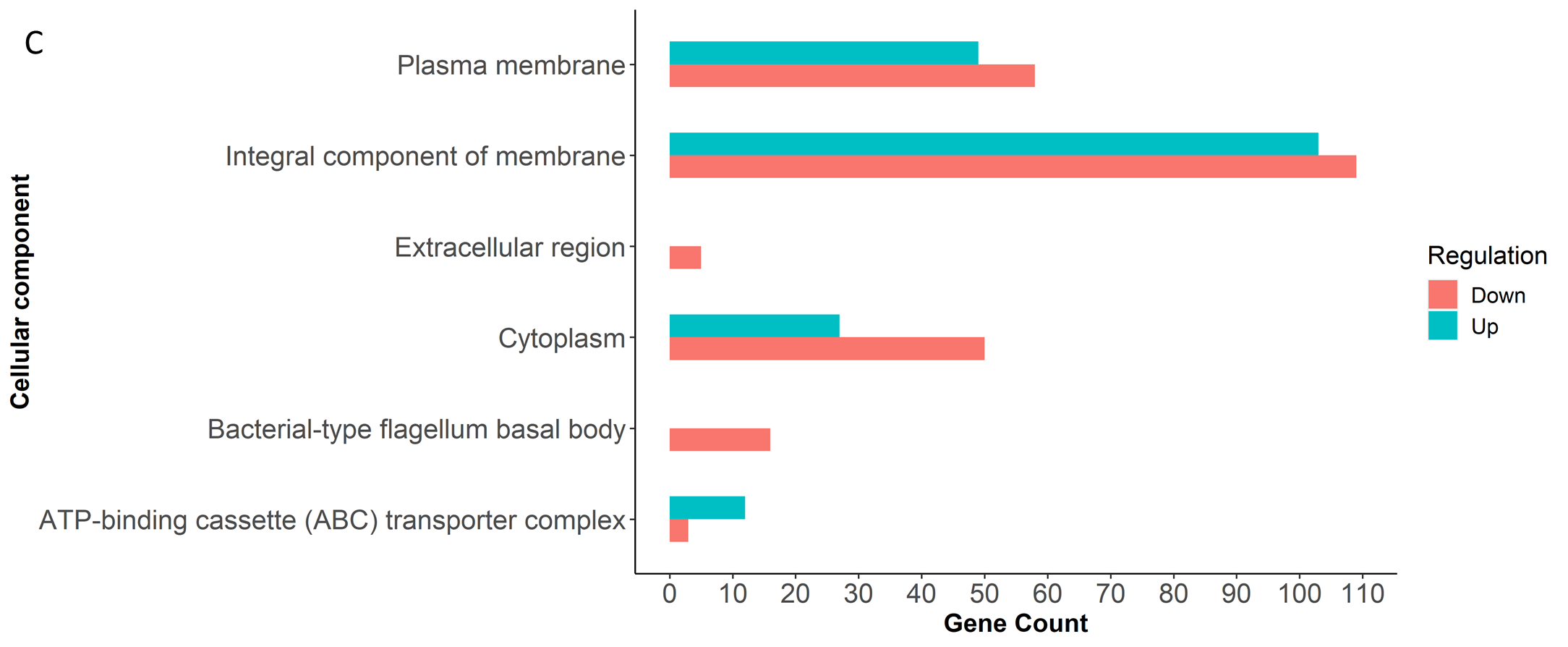

Supplement: Supplementary file 1 [file ijms-23-01396-s001.zip › Figure S4C.png]

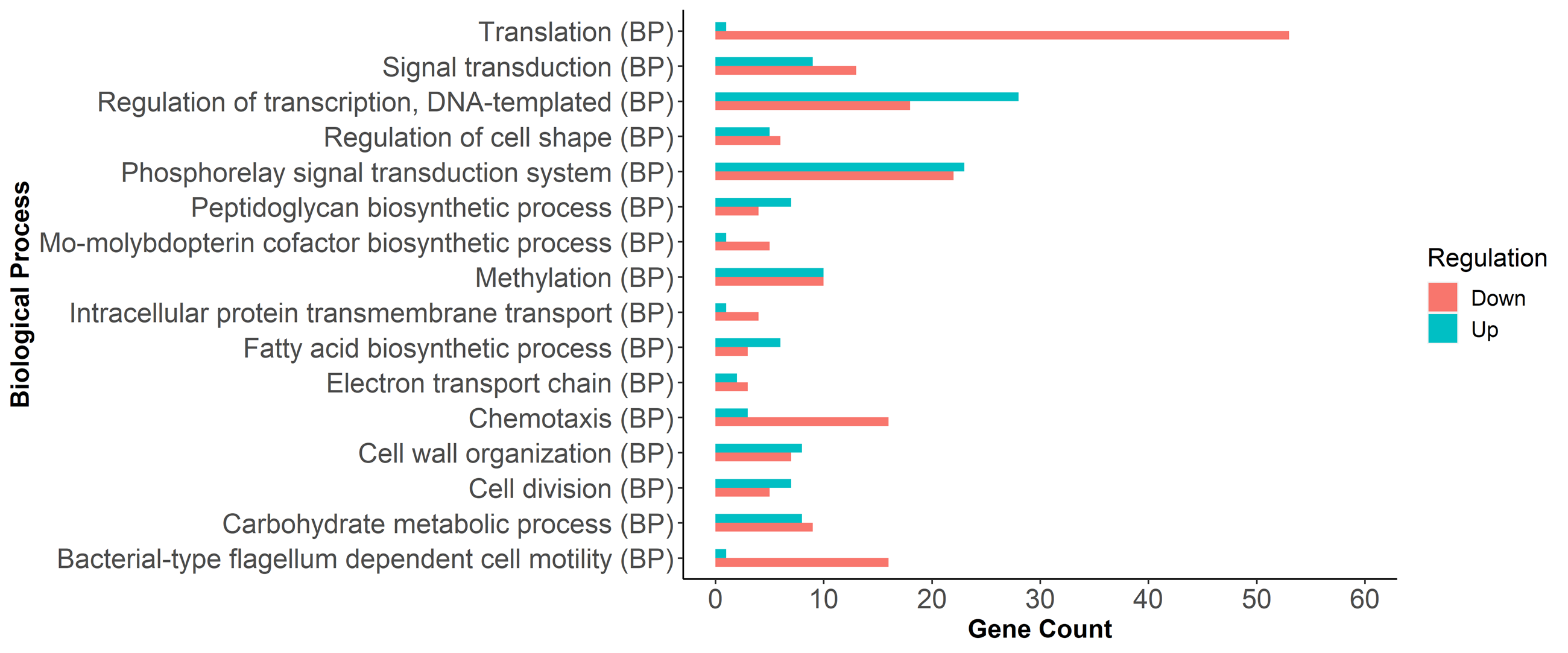

Supplement: Supplementary file 1 [file ijms-23-01396-s001.zip › Figure S4D.png]

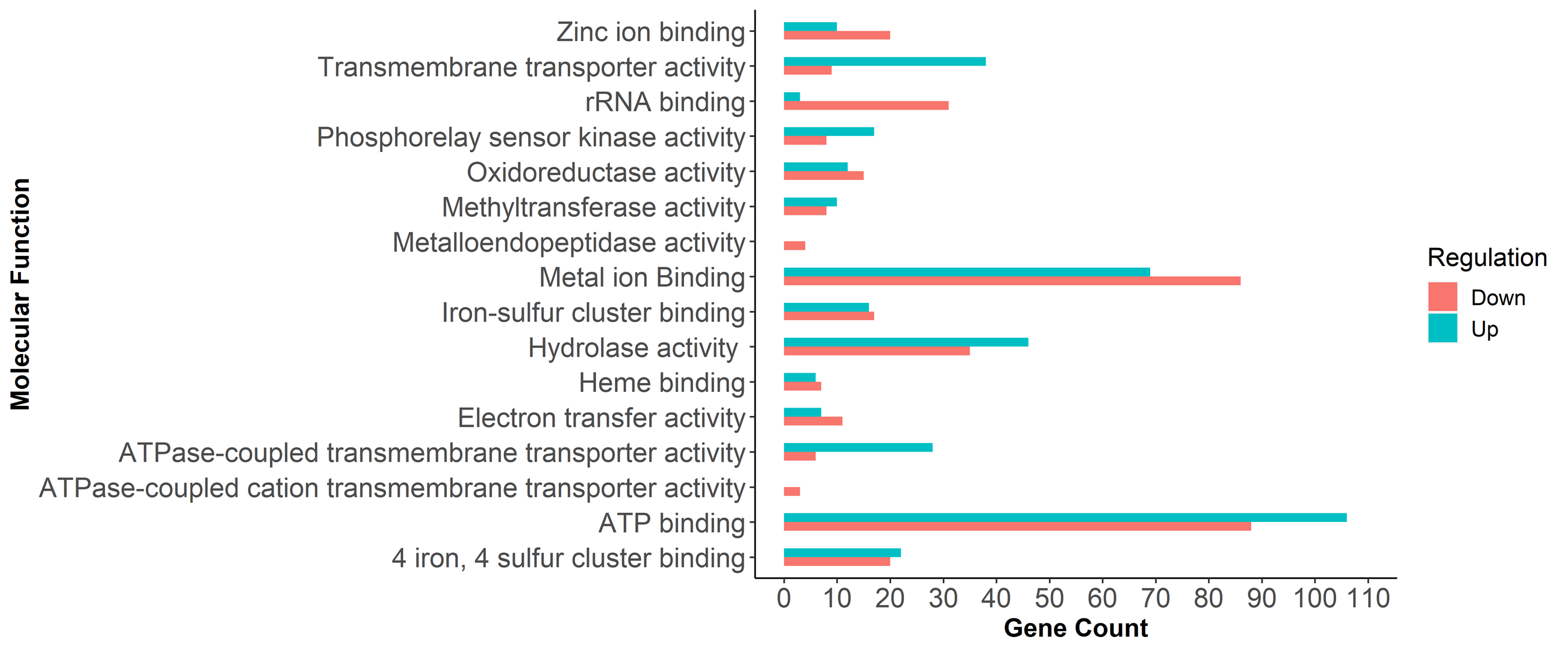

Supplement: Supplementary file 1 [file ijms-23-01396-s001.zip › Figure S4E.png]

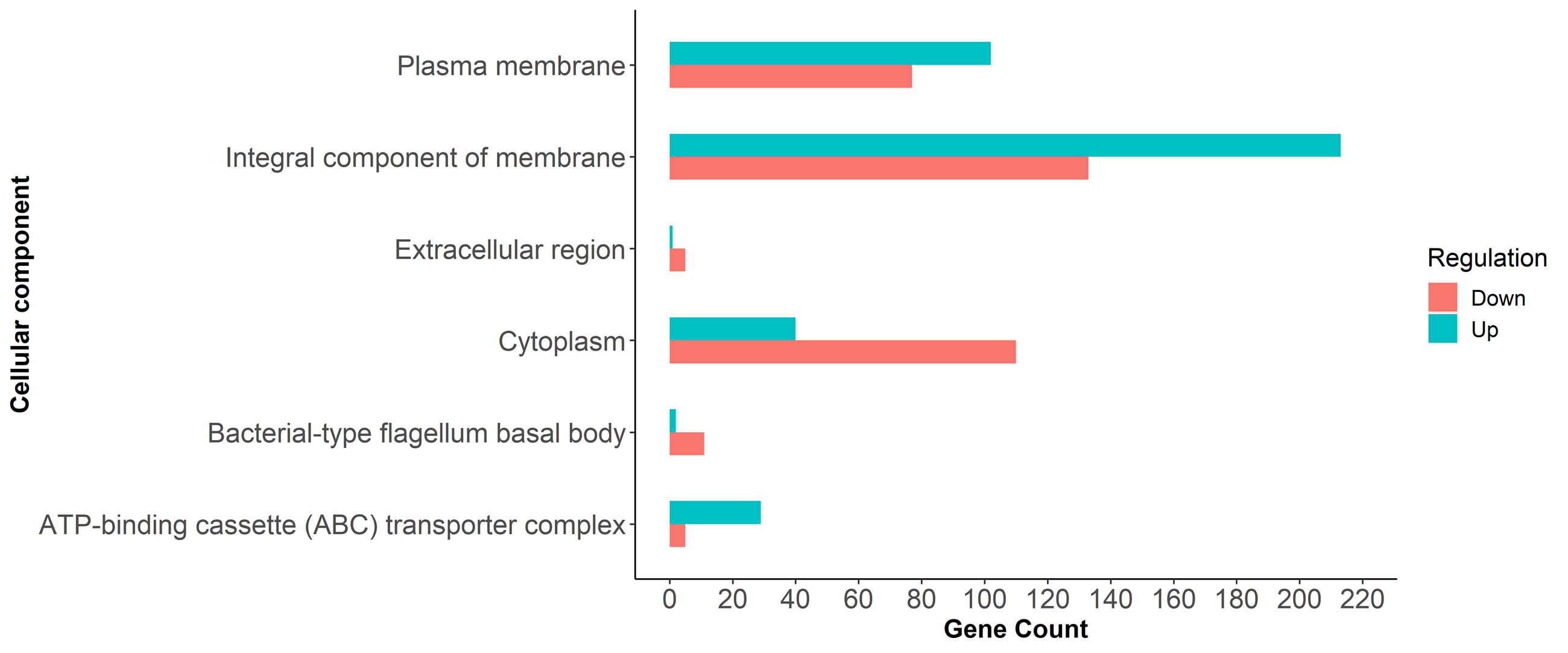

Supplement: Supplementary file 1 [file ijms-23-01396-s001.zip › Figure S4F.png]
